# Induction of functional Brm protein from Brm knockout mice

**DOI:** 10.18632/oncoscience.153

**Published:** 2015-04-18

**Authors:** Kenneth W. Thompson, Stefanie B. Marquez, Li Lu, David Reisman

**Affiliations:** ^1^ Division of Hematology and Oncology, Department of Medicine, University of Florida, Gainesville, Florida, USA; ^2^ Department of Pathology, University of Florida, Gainesville, Florida, USA

**Keywords:** brahma (BRM), SWI/SNF, lung cancer, knockout mice, aberrant splicing

## Abstract

Once the knockout of the *Brm* gene was found to be nontumorigenic in mice, the study of BRM's involvement in cancer seemed less important compared with that of its homolog, *Brg1*. This has likely contributed to the disparity that has been observed in the publication ratio between BRG1 and BRM. We show that a previously published *Brm* knockout mouse is an incomplete knockout whereby a truncated isoform of Brm is detected in normal tissue and in tumors. We show that this truncated Brm isoform has functionality comparable to wild type Brm. By immunohistochemistry (IHC), this truncated Brm is undetectable in normal lung tissue and is minimal to very low in *Brmnull* tumors. However, it is significant in a subset (~40%) of *Brg1/Brm* double knockout (DKO) tumors that robustly express this truncated BRM, which in part stems from an increase in *Brm* mRNA levels. Thus, it is likely that this mutant mouse model does not accurately reflect the role that Brm plays in cancer development. We suggest that the construction of a completely new mouse *Brm* knockout, where Brm is functionally absent, is needed to determine whether or not Brm is actually tumorigenic and if *Brm* might be a tumor suppressor.

## INTRODUCTION

The role of SWI/SNF in cancer was first realized with studies in the mid-1990s on the BAF47 (SmarcB1) subunit, which was found to underlie the genesis of Rhabdoid sarcoma [1-4]. Studies of this rare pediatric sarcoma led to the discovery of a 22q11 chromosomal re-arrangement that deletes the *BAF47* gene [5, 6]. Cell line studies have shown that the re-expression of BAF47 causes growth arrest [7], while targeted conditional knockout of *Baf47* in mice causes Rhabdoid tumor development when one or both *Baf47* alleles are inactivated [8-10]. Based on these data, BAF47 was determined to be a *bona fide* tumor suppressor. However, since the SWI/SNF complex is composed of 8-10 proteins, the fact that one of these proteins (BAF47) was a tumor suppressor [11, 12] led Bernard Weissman and others to hypothesize that other SWI/SNF subunits might also function as tumor suppressor proteins. Hence, investigators began to investigate the functional role of these other subunits in cancer, with a focus on the mutually exclusive SWI/SNF ATPases Brahma (BRM) and Brahma Related Gene 1 (BRG1)[13-15]; this opened the door to many new discoveries as to the role of chromatin remodelers in cancer. In the last several years, it has been shown that other SWI/SNF complex subunits appear to be frequently altered in human cancers. Specifically, NextGen sequencing studies have shown that mutations occur in the SWI/SNF subunits BAF180 and BAF250 in renal, gynecological and breast cancers [16-23], which suggests that these subunits are targeted during cancer [24]. The true impact of these various newly discovered SWI/SNF subunit mutations is unknown, as effective mouse knockout models have yet to be generated.

The power of BRM and BRG1 to hydrolyze ATP is a prerequisite for the mechanical function of SWI/SNF: specifically, this allows for the movement of histones along the chromatin, and therefore, the loss of BRG1 and BRM expression negatively impacts the function of this complex [25-27]. To this end, both BRG1 and BRM are often found to be individually and/or mutually silenced in many human cancer types [14, 15]. Initially, BRG1 and BRM were linked to cancer because of their critical associations with key cellular proteins including BRCA1, p53 and Rb [7, 14, 28-32]. In particular, a number of labs have shown that BRG1 and BRM actually bind to Rb and that the loss of BRG1 and/or BRM impairs Rb-mediated growth inhibition [33-35]. Moreover, like BAF47, the re-expression of BRG1 or BRM efficiently inhibits cell growth in BRG1/BRM-deficient cell lines [33, 34]. These data clearly link both BRG1 and BRM to cancer. However, in order to be classified as a tumor suppressor protein akin to BAF47, their inactivation in mice would ideally cause the formation of tumors. While BRG1 and BRM appear to be critical anticancer proteins based on *in vitro* data, their individual knockouts have not robustly induced cancer in recipient mice [36-38]. A complete knockout of *Brg1* was first found to be embryonically lethal [37]. This is not surprising since BRG1 is required for the expression of genes like beta hemoglobin along with other proteins that are essential for normal development and differentiation [39-47]. The heterozygous inactivation of *Brg1*, however, produces mice that are viable but that invariably develop breast-like tumors within 1 year [38]; in murine lung cancer models, a lung-specific carcinogen combined with Brg1 inactivation significantly potentiates lung cancer development [48]. Hence, Brg1 inactivation appears to be moderately tumorigenic. In contrast, the knockout of Brm produces viable mice, but the loss of Brm is not tumorigenic [36]. Since Brg1 appears to be tumorigenic and Brm does not, this greatly diminishes the perceived importance of BRM in cancer development [37, 38]. While not tumorigenic, *Brmnull* mice are reported to be bigger than wild type mice, and mouse embryonic fibroblasts (MEF) from *Brmnull* mice demonstrate abnormal cell cycle control [36]. Consistent with this finding, we have reported that the *Brmnull* phenotype potentiates cancer development when combined with carcinogens, which indicates that BRM is not a tumor suppressor, but rather, a tumor susceptibility gene [49]. As such, we hypothesized that since BRG1 and BRM are homologs, the loss of one could be compensated for by the expression of the other. This is supported by the finding that re-expression of either gene in BRG1/BRM-deficient cell line results in the induction of similar genes such as *CD44* and *CSF-1*, among others [50]. Moreover, the expression of either BRG1 or BRM has been found to be sufficient to cooperate with Rb to foster Rb-mediated growth inhibition [14, 51]. The observed functional redundancy of BRG1 and BRM may explain why BRG1 and BRM are both found to be lost in aggressive cancers [50]; the silencing of one of these genes only partly abrogates SWI/SNF function while their combined loss completely blocks any SWI/SNF function and thus, is likely more tumorigenic [50]. As the individual loss of either BRG1 or BRM is highly tumorigenic, we hypothesized that the loss of both BRG1 and BRM completely inactivates SWI/SNF-dependent pathways, which causes cancer development. To test this hypothesis, we used our murine lung tumor model to determine the impact of the inactivation of Brg1, Brm or both on the development of lung tumors compared with wild type mice [48, 49] (unpublished data). Surprisingly, a subset of tumors derived from *Brg1/Brm* DKO mice was observed to readily express Brm protein by immunohistochemistry (IHC). We investigated this paradoxical observation and found that the targeted Neo construct, which inactivates exon 4, is a perfect triplicate, which means that splicing around this exon left the transcript in-frame. This hybrid *Brm* mRNA is transcribed at low levels compared with the wild type *Brm* mRNA, and thus, the resultant truncated BRM protein is marginally detectible via western blot in normal tissue. However, in a subset of tumors derived from *Brg1/Brm* DKO mice, the *Brm* transcript was increased to ~8 delta Ct values, so that BRM protein expression was readily detectible by IHC. We therefore hypothesize that the *Brmnull* mouse model that was used in these studies is not a true knockout and that conclusions about whether or not BRM is a tumor suppressor must await the production of a genuine *Brm* knockout.

## RESULTS

### Brg1- and Brm-deficient tumors paradoxically express Brm

**Figure 1:**
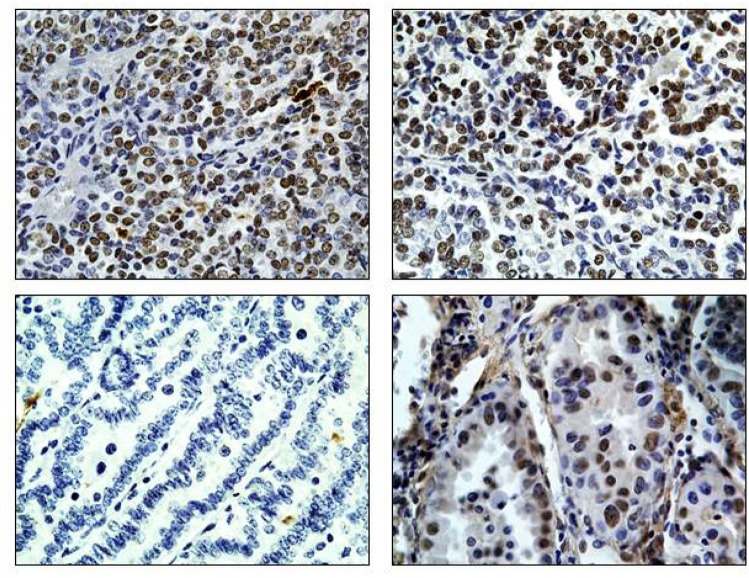
Expression of Brm in *Brg1/Brm* double knockout mouse lung tumors Using a BRM-specific antibody, immunohistochemical staining shows the distribution of Brm protein expression in wild type mice (top left), *Brmnull* mice (top right), and *Brm/Brg1* double knockout tumors (bottom right). A *Brmnull* tumor with no BRM staining is also shown (bottom left). All images were obtained at 63x.

The loss of Brg1 or Brm alone in mice has been found to be weakly tumorigenic or not tumorigenic, respectively [36-38]. As BRG1 and BRM are often simultaneously lost in human cancer [14, 15, 52], we developed a double knockout murine system to determine if the loss of Brg1 together with loss of Brm might be more tumorigenic than the loss of either one alone. As part of the initial analysis, we stained each of the resultant Brg1- and/or Brm-deficient tumors. We found that tumors derived from wild type and Brg1-only knockout mice were both readily immunopositive for Brm by IHC as expected based on the genotype, (Figure 1). Interestingly, we found that a subset of the tumors from *Brg1/Brm* DKO mice stained positive for Brm (Figure 1; Table 1). Moreover, the *Brmnull* mice tumors were not always negative as anticipated by their genotype. But rather, a subset of these tumors demonstrated faint or weak Brm staining. In order to evaluate the expression of Brm in DKO mice compared with wild type mice, we used a standard scoring system where the intensity of staining was scored 0-3 and the percentage of positive cells was scored 0-100%. Based on the product of the intensity and the percentage of positive cells, each tumor was assigned a product score where 0-50 indicates no expression, 50-100 indicates low expression, 100-200 indicates moderate expression, and 200-300 indicates high expression. We found that wild type and DKO gave the following percentages in these four categories, respectively: WT 0%, 15.4%, 38.5%, and 46.1% and DKO 50.0%, 31.2%, 12.5%, and 6.7% (Table 1). Comparatively, the vast majority (~85%) of *Brmnull* tumors received scores of 0-50, and ~15% scored in the low expression category (Table 1).

**Table 1:** The percentage of wild type, *Brmnull* or DKO tumors that demonstrated very low, low, moderate or high levels of BRM protein expression by IHC

	Wild type (n=13)	*Brmnull* (n=13)	DKO (n=16)
Very low	0.00%	85.00%	50.00%
Low	15.38%	15.00%	31.20%
Moderate	38.46%	0.00%	12.50%
High	46.15%	0.00%	6.70%

### Brm mRNA Undergoes Alternate splicing and bypasses the targeted exon

**Figure 2:**
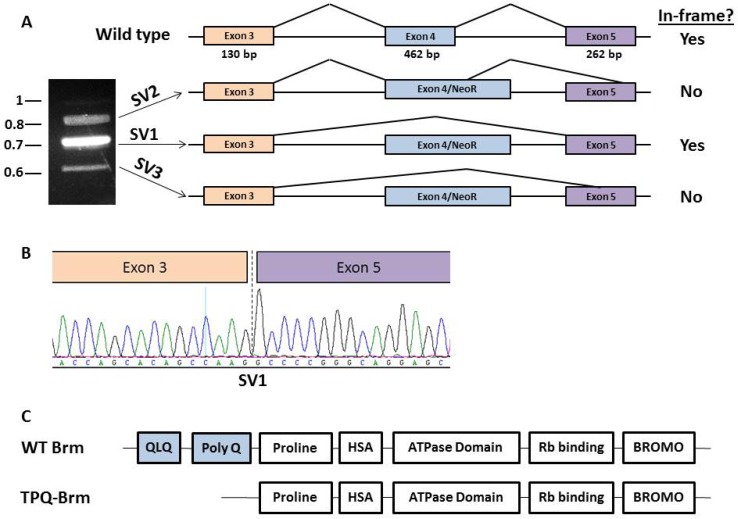
Identification of an in-frame truncated *Brm* transcript in *Brmnull* mice A. Agarose gel shows three splice variants isolated from the lung cDNA pool of a *Brm* knockout mouse, and depiction of *Brm* transcripts shows alternative splicing around exon 4. An image of this agarose gel shows three PCR products amplified from the lung cDNA pool with primers that flanked exon 4: 812 bp (SV2), 711 bp (SV1), and 623 bp (SV3), respectively. Arrows depict splicing around exon 4 for each PCR product. Exons are shown as black boxes and are numbered according to the accession number NM_011416.2. The top figure depicts wild type conditions where exons 3, 4, and 5 (sizes are given) are properly spliced together, while the bottom three drawings illustrate the three splice variants that were observed. The SV2 variant results from aberrant splicing from within molecularly modified exon 4-neomycin (Exon 4/NeoR) to within exon 5 at base pair 256. The SV1 variant results from splicing around exon 4/NeoR, which then splices normally to the 5′ end of exon 5, while SV3 also splices around exon 4 but then splices into the middle of exon 5 at bp 131 of exon 5. The right side of the figure shows whether or not each spliced transcript results in an in-frame *Brm* transcript. B. A chromatogram depicts the deletion of exon 4 in splice variant 1. The end of exon 3 and the beginning of exon 5 are shown, and the dashed line marks where exon 4 is deleted. C. The conserved domains in both WT Brm and TPQ-Brm protein are shown. This figure also illustrates how the omission of exon 4 in TPQ-Brm results in the loss of the PolyQ domain (light blue in A).

To unravel this seemingly paradoxical result where Brm expression can be observed in the *Brg1/Brm* DKO genotype, we re-examined the generation of the BRM-targeted mice described in Reyes et al. [36]. We identified the targeted exon as exon 4 (by comparison with the cDNA NM_ 011416.2) and noted that its length of 462 base pairs is an even triplicate such that its omission does not cause a frame shift (Figure 2A). Hence, we predicted that splicing around this targeted exon would shorten the resultant protein 154 amino acids (from 1590AA to 1436AA) but would not cause a frame shift or the loss of protein expression. To demonstrate this, we conducted PCR with primers that flanked exon 4 and observed a band that was indicative of an mRNA fragment that lacked exon 4 (middle band in Figure 2A). The sequencing of this PCR product, called splice variant 1 (SV1), showed that the 462 bp corresponding to exon 4 were absent (Figure 2A and B). Additionally, two other splice variants, called splice variant 2 (SV2) and splice variant 3 (SV3), were observed by PCR. Sequence analysis showed that each of these two transcripts resulted in a frame-shifted *Brm* transcript and a nonfunctional Brm protein. By sequencing the SV2 (upper band in Figure 2A), we observed the expected molecularly modified (targeted) *Brm* exon 4 where the first 198bp of *Brm* exon 4 is present, followed by the replacement of the latter 264bp of exon 4 with the *neomycin* gene. The reading frame of this resultant *Brm* transcript creates a stop codon within the *neomycin* gene and in turn, a truncated, nonfunctional Brm protein. Additionally, we observed a third transcript (SV3) that splices from the end of exon 3, bypasses exon 4 and splices aberrantly near the middle of exon 5 (bottom band in Figure 2A). This third transcript also results in a frame-shifted transcript and a nonfunctional Brm protein. Therefore, although the two splice variants that are present lead to truncated Brm proteins, we identified one transcript variant that yields an in-frame transcript and a nearly full-length Brm protein. This occurred because this particular transcript can splice around exon 4, and since exon 4 is a perfect triplicate, the splicing variant remains in-frame.

### Loss of the PolyQ domain

The BRM protein has several unique domains, but only one is clearly essential: the helicase domain that converts ATP energy into mechanical energy [53]. This domain is essential for BRM function, as a dominant-negative isoform of BRM can be created by the introduction of a missense mutation into the contact point with ATP, which prevents the metabolism of ATP by BRM [53]. Other definable domains of BRM are the “Bromo” domain, and “Rb-binding: LXCXE” domain, which bind acetylated histone and the Rb protein, respectively [54]. While the loss of the Bromo domain appears to impact the regulation of gene expression, the loss of the Rb-binding domain has been shown to block Rb-mediated growth inhibition [35]. Other domains, such as the QLQ, PolyQ, proline-rich and HSA that are located near the N-terminus, are not yet functionally understood [53, 54]. As the QLQ and PolyQ domains are located in exon 4, these domains are absent in the Brm splice variant 1 (Figure 2C). While some proteins with PolyQ domains are known to undergo expansion of this region by DNA polymerase stuttering, no examples of BRM inactivation due to expansion have been documented thus far [49]. In fact, the sequencing of a variety of cancer cells and lung tumors has shown that the BRM PolyQ domain appears to remain essentially invariant (unpublished data)[49]. Thus, in the identification and purification of this Brm isoform, we had the added benefit of assessing the potential functionality of the PolyQ and QLQ domains. In this case, all thirty-nine glutamines were present within exon 4 so the entire QLQ (6 glutamines) and PolyQ (33 glutamines) region was removed. We will henceforth refer to this isoform as Truncated PolyQ Brm or TPQ-Brm.

### Truncated Brm protein is functional

**Figure 3:**
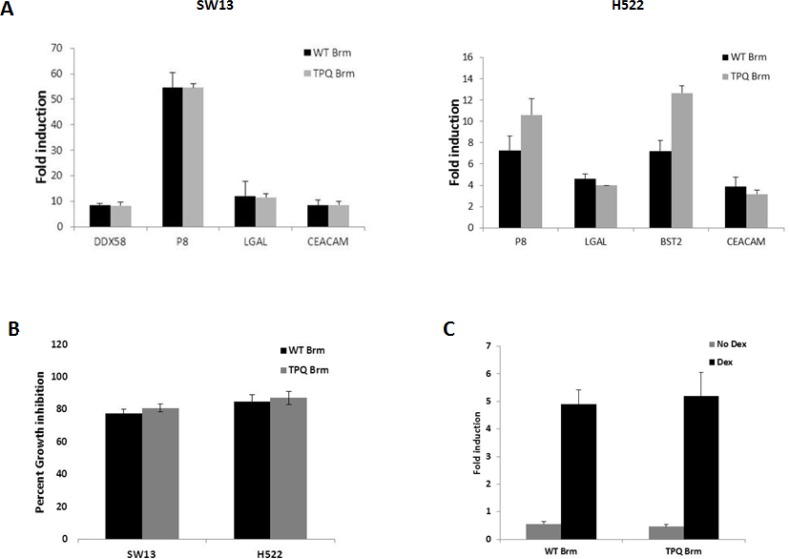
TPQ-BRM has a similar functionality to wild type BRM A. The BRG1/BRM-deficient cancer cell lines SW13 (left) and H522 (right) were transfected with wild type *Brm*, TPQ-*Brm*, or empty vector; *Brm-*dependent gene induction was then measured by qPCR. In both SW13 and H522 cell lines, these *Brm-*dependent genes were similarly induced by wild type and TPQ-*Brm* compared with the empty vector control. B. Introduction of either WT *Brm* or TPQ-*Brm* causes significant growth inhibition. SW13 or H522 cells were infected with lentivirus containing either empty-, wild type *Brm*- or TPQ-*Brm*-pCDH-EF1-GFP vectors. By measuring the number of GFP expressing cells daily for 7 days, the percent growth inhibition for wild type Brm and TPQ-*Brm* compared with the GFP empty vector control was calculated. C.SW13 cells were co-transfected with either empty vector, wild type *Brm*, or TPQ-*Brm* in pCDH-EF-1-GFP, along with pcDNA3.1-GR, pMMTV-luc reporter and the Renilla plasmid, which was used for normalization. Following transfection, cells were treated with 10-7 M Dexamethosone or vehicle control. Ethanol and luciferase activity was measured 24 hours later. The fold induction of luciferase activity of *Brm* or TPQ-*Brm* relative to the empty vector control with or without Dexamethasone is shown. The error bars represent the standard error of the mean of triplicate experiments.

Given that Brm is not inactivated in the exon 4 knockout design, an important question is whether this truncated Brm is functional. To investigate whether the TPQ-Brm protein is functional and could nullify the intended *Brmnull* tumor phenotype, we conducted several functional assays to compare wild type and TPQ-Brm. First, both wild type and TPQ-*Brm* were cloned and inserted into pCDH-EF1-GFP expression plasmids for functional analysis. As BRM and SWI/SNF regulate a variety of genes, we previously conducted microarray experiments and identified a number of BRM-dependent genes which were then verified by quantitative RT-PCR (qPCR) [55, 56]. We transfected wild type *Brm* or the TPQ-*Brm* plasmids into two BRM/BRG1-deficient cell lines, SW13 and H522, and examined these two transfected cell lines for the induction of four genes (SW13: *DDX58*, *P8*, *LGALS3*, and *CEACAM-1*) and (H522: *P8*, *LGALS3*, *BST2*, and *CEACAM-1*). These genes were selected because they have been shown to have a role in disease and/or cancer development. As such, the human *DDX58* gene product is a viral sensor for infection [57], the human *P8* gene is a putative tumor suppressor that controls metastatic behavior [58], the human *LGALS3* gene is a marker of tumor progression [59], the human *BST2* gene is overexpressed in breast cancer and is involved in tumor metastasis [60], and finally, the human *CEACAM-1* gene is involved in angiogenesis in non-small cell lung cancer [61]. qPCR analysis showed that both wild type Brm and TPQ-Brm induced similar expression levels of these known BRM dependent genes in the BRM/BRG1- deficient cell lines (Figure 3A). Thus, the TPQ-Brm appears to be functional with respect to gene regulation.

We next examined BRM-mediated growth inhibition. Elegant experiments performed by Strober and Duniaef showed that BRG1 and/or BRM-mediated growth inhibition depends mostly on Rb and to lesser degree on the Rb homologs RB2 (p130) and p107 [33, 34]. Conversely, several labs have shown that Rb fails to inhibit growth when introduced into BRG1/BRM-deficient cell lines, while Rb-mediated growth inhibition is restored when either BRG1 or BRM is co-expressed with Rb [14, 51]. We therefore made lentivirus to the wild type Brm or TQP-Brm constructs cloned into the pCDH-EF1-GFP lentiviral vector. Empty vector, BRM- or TQP-BRM-containing virus was used to separately infect SW13 and H522 cells; GFP-based cell counts were conducted daily for 5-7 days, and then the growth rate was calculated for each condition. These experiments showed that the degree of growth inhibition was not significantly different between Brm and TQP-Brm compared with empty vector; both the Brm and TQP-Brm demonstrated approximately 80% growth inhibition when introduced into the BRM-deficient cell lines SW13 and H522 (Figure 3B). Interestingly, we found that the TPQ-Brm variant retained its ability to partially induce differentiation, as we observed that SW13 cells infected with TPQ-Brm exhibited a flattened or “fried egg” appearance, which has been described by other investigators when either BRG1 or BRM was introduced into SWI13 cells [33, 34, 62]. These data indicate that TQP-Brm and Brm are functionally similar with respect to growth inhibition.

SWI/SNF and BRM are known to potentiate the function of steroid receptors, in particular the glucocorticoid receptor (GR) [63, 64]. To this end, we utilized a GR-dependent assay that was previously used to demonstrate SWI/SNF and BRG1/BRM function [65]. In this assay, the ligand-activated steroid receptors (i.e., androgen, progesterone, or estrogen) bind to an inducible MMTV-promoter, which then drives the expression of the luciferase reporter gene (MMTV-Luc). We transfected this MMTV-Luc reporter construct along with Rat GR into the BRG1/BRM-deficient cell line, SW13; in order to standardize the transfection efficiency, we also transfected a plasmid that expresses the *Renilla luciferase* gene. Since the GR is SWI/SNF-dependent, the expression of BRM restores SWI/SNF function and the ability of GR to drive luciferase expression from this reporter. Hence, the output of luciferase is an indirect measure of BRM functionality. In this assay, we co-transfected the empty vector pCG, pCG-Brm or pCG-TQP-Brm, along with the rat GR expression plasmid, and the pMMTV-luc reporter plasmid into the BRG1/BRM-deficient cell line, SW13. After 48 hours, these SW13 cells were then treated with either the GR ligand Dexamethasone or the carrier, ethanol, as a negative control. After an additional 24 hours, we measured and observed that compared with empty vector, both Brm and TQP-Brm induced luciferase expression approximately equally (Figure 3C). Taken together, these data demonstrate that TQP-Brm is functional with respect to fostering GR transcription.

### TPQ-Brm mRNA levels in normal tissue versus tumor tissue

**Figure 4:**
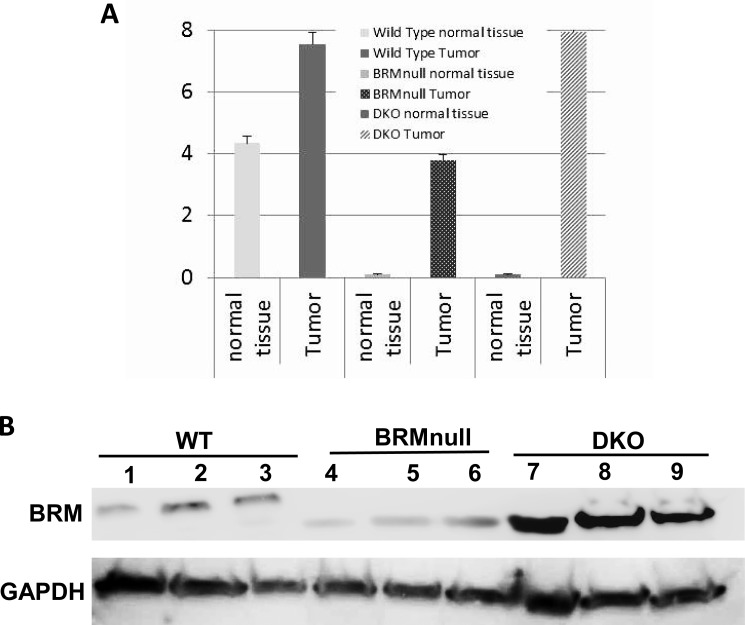
Brm expression in double knockout tumors is elevated in mouse tumors **A.** Comparison of *Brm* mRNA levels in tumors by qPCR analysis. **B.** Western blot showing Brm protein expression for three tumors each for the wild type, *Brmnull* and double knockout genotypes. To emphasize the induction of BRM in the DKO, we selected the three tumors with the highest BRM expression by IHC and three tumors from *Brmnull* and wild type mice which had a median level of BRM expression for each genotype.

Although it lacks the PolyQ domain, the TPQ-Brm appears to have similar functionality to wild type Brm as exemplified by the above *in vitro* assays. To determine if changes in the *Brm* mRNA levels underscored this observed increase in BRM protein, we examined *Brm* mRNA levels in normal tissue and in tumors from wild type, *Brmnull* and *Brm/Brg1* DKO genotypes. We found that the mRNA levels in *Brmnull* and DKO mice were approximately 4.3 delta Ct values lower (approximately 5%) than in wild type mice (Figure 4A). This is consistent with the 1% of *Brm* mRNA levels in the *Brmnull* mice reported by Reyes et al [36]. We next examined the levels of *Brm* mRNA in normal tissue compared with adenocarcinomas from each of the three genotypes. We observed that the mRNA levels increased by 3.5, 4, and 8 delta Ct values for the wild type, *Brmnull*, and double knockout, respectively (Figure 4A).

The impact of these changing mRNA levels can be illustrated by a western blot of wild type, *Brmnull* and DKO tumors (*Brg1/Brm*-deficient). Although Brm proteins can only be readily detected in wild type and some DKO tumors by IHC (Figure 1), the increased sensitivity of western blotting allows for the detection of the Brm protein in tumors from all three genotypes (Figure 4B). After an examination of the western blot data, it is clear that *Brmnull* tumors have significantly less Brm protein than the other two genotypes. Moreover, the increases in *Brm* mRNA in tumors from DKO animals are higher on average than the increases in the tumors from wild type animals. Lastly, the BRM protein that was purified from the *Brmnull* and DKO tumors is notably shorter (lower band by western, Figure 4B) compared with the Brm protein that was purified from the wild type tumors. Hence, by IHC and western blot, Brm proteins from the DKO mice are readily detectible, and in certain cases are expressed at a higher level, than Brm protein from wild type mice.

## DISCUSSION

The original data presented on the *Brm* knockout mouse model implied that Brm was functionally absent in the *Brmnull* mice [36]. During their work, Reyes et al. [36] did actually observe a smaller *Brm* splice variant that spliced around the targeted exon (exon a in Reyes *et al*. and exon 4 here); however, they did not observe the TPQ-Brm protein via western blot, which we detected in our study. This difference is likely due to the fact that we examined BRM expression in murine lung tumors while they examined BRM expression in normal tissues where expression levels are much lower and thus difficult to detect. However, they could detect Brm after they enriched for TPQ-Brm protein by first conducting Brm-specific immunoprecitation followed by western blot, where a smaller, faint band was detected from protein extracts from *both* wild type and *Brmnull* mice (Figure 2E in Reyes et.al) [36]. In addition, in their experiments, the BAF155 and BAF47 proteins only co-immunoprecipitated from the wild type mouse tissue extracts, but not from the *Brmnull* tissue extracts; they concluded that due to the lack of communal binding of these SWI/SNF subunits, that the TPQ-Brm must lack functionality. One caveat to this experiment is that the smaller protein band clearly appears in both wild type and *Brmnull* lanes, which implies that this *Brm* splicing variant might occur naturally. Another caveat to this experiment is that the diminished TPQ-Brm protein levels would be expected to pull down much less BAF155 and BAF47 protein, possibly below the limits of detection by western blot. That the TPQ-Brm was only barely detectable in this experiment supports this idea. Moreover, as polyQ functions to enhance protein interaction, the lack of this domain may have weakened Brm interactions with other SWI/SNF subunits so that the results of these co-IP experiments may have been due to reduced protein binding of complex components rather than a lack of Brm protein. To this end, Reyes et al. determined that the TQP-Brm was <1% of the total Brm in wild type cells [36]. Hence, the question becomes whether or not the lower levels in normal tissue impart a different phenotype than that which is seen in malignant cells. To this end, Reyes et al. did report the observation of alterations in cellular growth control in the *Brmnull* mice and murine embryonic fibroblasts (MEFs) which makes sense, as lower levels of Brm would be expected to partially impair the function of RB and Rb2 (p130). Based on their reported data, one cannot determine if these Brm levels are sufficiently low so as to marginally impair growth control, while the complete absence of Brm might actually be tumorigenic. Hence, the lack of a truly complete *Brm* knockout murine model makes any absolute assertions about the lack of tumorigenicity of Brm nearly impossible.

Since Reyes published that the knockout of *Brm* in mice was not tumorigenic, this conclusion had a rippling effect in regard to the perception of the BRM protein and its role in cancer development. This situation is highlighted by the fact that there have been only 98 publications as of March 2015 on the biology of BRM (in the title) compared with 241 publications that have focused on the biology of BRG1, even though BRG1 and BRM are highly homologous proteins with similar cellular functions. Thus, the bias in this publication ratio suggests that the scientific community perceives BRG1 as a more relevant cancer gene (protein) than it does BRM. However, the knockout of BRM in BRG1-deficient cell lines has led to the realization that BRM is important for growth, and its loss has been shown to impede cellular proliferation [66-68]. To this end, SWI/SNF and BRM have been found to facilitate the proliferative function of oncogenes such as c-Myc and c-Jun [69-72]. Unfortunately, we probably will not know the true tumorigenic potential of BRM loss until a true *Brm* knockout is constructed and characterized. Ideally, putative *Brm* knockouts should also be tested with and without *Brg1* knockout to eliminate the potential compensatory function of Brg1 on Brm and vice versa. From our own experiments, it is clear that a certain fraction of tumors that are derived from DKO (*Brg1* and *Brm*) mice have elevated Brm expression and that the resultant tumors are likely phenotypically similar to tumors that arise in the Brg1 knockout phenotype (where Brm expression is retained).

Our findings suggest that the ability of the *Brm* gene to splice around the damaged exon is a compensatory mechanism in this murine system. Our data indicate that the loss of the PolyQ region in the *Brm* gene, which has approximately 35 glutamines in a row, does not appear to inhibit or block BRM function as measured by *in vitro* assays. This result is not surprising since BRG1 is functional in the absence of the PolyQ domain; this is the only one of several major functional domains that is not conserved between BRM and BRG1 [50]. As noted, PolyQ regions are often associated with expansion and then dysfunction of the resultant protein, but there is no evidence yet that suggests that the *BRM* gene undergoes expansion. In fact, PolyQ tracts in proteins are thought to stabilize protein interactions [73], which makes sense since BRM is part of a multimeric complex of 10 other proteins. To-date, an analysis of these PolyQ-containing proteins has shown that these regions are found in approximately 137 different proteins [74, 75]. The study by Muchardt and Yaniv is essentially the only study that has examined the impact of BRM domains on transcription [53]. These investigators found that the deletion of the PolyQ, Proline-rich and Charged Regions decreased the transcriptional activity of the GR by ~65% [53]. However, in our assay, the deletion of the PolyQ region did not significantly affect the functionality of TPQ-BRM. Hence, it is likely that either the Proline-rich or Charged region alone or the combined loss of the PolyQ plus the Proline-rich areas play a role in transcriptional activation. As such, a more detailed analysis of these regions will be needed to specifically delineate the function of the PolyQ domain in conjunction with these other specific regions within the *BRM* gene.

In summary, the current published *Brm* knockout mouse model leads to mice that express low but detectible levels of functional Brm protein. As such, the impact of these low levels of Brm, as well as the higher levels that can be potentially induced, indicate that the true impact of an *in vivo Brm* knock out (inactivation) is not yet known. However, studies in cancer patients have shown that BRM loss appears to affect cancer development [15, 76-78]. Based on data derived from case-controlled studies on the presence of the BRM promoter polymorphisms, BRM loss can be indirectly linked to cancer development as well as inferior clinical outcomes [79-83]. Moreover, the fact that BRM expression can be induced or activated by deacetylation by the vast majority of, if not all, Flavonoids attests to the importance of BRM in cancer development [84]. In addition, Rb and p53 dependency on this gene also indicate a role for BRM loss in cancer development [14, 28, 30, 34]. BRM is located in an area of reported loss of heterozygosity, and BRM loss occurs in a significant portion of adult human solid tumors, including a vast majority of Rhabdoid tumors [85]. Whether *BRM* is a tumor suppressor gene or a tumor susceptibility gene, as suggested by the *BRM* promoter polymorphism data, must await the development and characterization of a complete *Brm* knockout model.

## MATERIALS AND METHODS

### Isolation of Brm splice variants from lung cDNA and sequencing

RNA from lung tissue was extracted from a *Brmnull* mouse using an RNA isolation kit from Sigma-Aldrich (#RTN350) according to the manufacturer's instructions. For cDNA production, the SuperScript III First-strand kit from Life Technologies (#18080-051) was used. A nested PCR approach was used to amplify the *Brm* splice variants using *Brmnull* lung cDNA with forward primer 5′-GGAAGATTCAGCCAGCACAC-3′ and reverse primer 5′-ATCAGCCTCCGCATTCTCT-3′ and then forward primer 5′-TAACTGGCAGAGCCAGGAGA-3′ and reverse primer 5′- AAATCTGGTGGCAAGGAACC-3′ under the following reaction conditions: 94°C for 3 minutes, 40 cycles of 94°C for 30 seconds, 63°C for 30 seconds, and 72°C for 2 minutes and 30 seconds. The PCR fragments were gel-extracted (Qiagen kit, #20021), cloned into pGEM-T Easy vector (Promega) and sequenced with universal M13 primers by Genewiz Inc. (South Plainfield, NJ, USA).

### Cloning of TPQ-Brm into pCG and pCDH-EF1- GFP plasmids and quantitative RT-PCR (qPCR) on BRM regulated genes and mouse tumors

The pCG-Brm vector has been described previously [36]. For construction of pCG-TPQ-*Brm*, the 711 bp murine BRM splice variant (Figure 2A) was cut out of the pGEM-T Easy vector and cloned into pCG-*Brm* via *XbaI* and *FseI* restriction sites. The wild type *Brm* and TPQ-*Brm* genes were cloned into pCDH-EF1-GFP (System Biosciences) using the *EcoRI* sites and the *XbaI* and *EcoRI* sites, respectively. Transfection and qPCR analysis of BRM regulated genes were performed as follows: SW13 or H522 cells were plated in 6-well plates and were transfected with 1.5 μg of either empty vector or pCDH-EF1-GFP containing wild type *Brm* or TPQ-*Brm* using JetPRIME transfection reagent (Polyplus, #114-75). After 72 hours, RNA was isolated and cDNA was produced as described above. For qPCR, an initial 10-minute denaturation step at 95°C was used followed by 40 cycles of 95°C for 10 seconds and 60°C for 30 seconds. Target gene levels were normalized to *POLR2A* and the delta delta Ct method was used to calculate the fold induction of BRM regulated genes compared with empty vector [86]. Mouse tumor cDNA was prepared as described above and *Brm* qPCR levels were normalized to *Gapdh*. qPCR primers are listed in supplemental table 1.

### Viral production, Growth inhibition assay and Luciferase reporter assay

In all, 10 ug of either empty vector or pCDH-EF1- GFP containing wild type *Brm* or TPQ-*Brm* and viral packaging vectors (Addgene vectors pMD2.G VSV-G (12259) and psPAX2 (12260)) were co-transfected into H293T cells. Both SW13 cells and H522 cells were infected twice with 20Xvirus for 24 hours each. Following infection, cells that expressed GFP were counted on an Accuri C6 (BD Biosciences) flow cytometer at days 5-7. The percent growth inhibition was calculated from the ratios of the growth curve slopes. For the luciferase assay, JetPrime transfection reagent was used to transfect cells with *Renilla luciferase* expression plasmid, the pMMTV-luc reporter plasmid, and the rat GR expression plasmid (pCDNA3.1-GR plasmids); these latter two plasmids were a kind gift from Jorge A. Iniguez-Lluhi at the University of Michigan. Firefly luciferase and Renilla luciferase activity was determined with the Dual-Luciferase reporter assay system (Promega #E1960) and read on an FLx800 plate reader (BioTek).

### Immunohistochemical Staining (IHC)

IHC was conducted as previously described [15, 49, 87]. Antigen retrieval for BRM was performed with 10mM Tris (pH 10) according to the manufacturer's guidelines. The anti-BRM rabbit antibody is described in Glaros et al. 2007 [49]. A goat anti-rabbit or goat anti-mouse biotinylated secondary antibody was used with these primary antibodies at 1:200 (Vector Labs). The sections were incubated with primary antibodies for 2 hours at room temperature and with secondary antibodies for 1 hour. We used an ABC staining kit with DAB/nickel detection reagent (BD Pharmingen, San Diego, CA, USA). Slides were counterstained with Harris hematoxylin for 2 minutes.

### Western Blotting

For western blots, mouse tumor lysates were extracted with Urea buffer (8.M Urea, 50mM NaH_2_ PO4, 150 mM NaCl, 0.5% NP-40, 1M Tris pH 8.0). A total of 80 μg of protein was mixed with 6x Lamelli buffer, boiled for 10 minutes and then loaded in a 4-15% Bio-Rad precast gel (Bio-Rad, Hercules, CA, USA). Gels were run for 1 hour at a constant voltage of 130 V. Subsequently, proteins were transferred to a Millipore Immobilon P membrane. Proteins were transferred for 1 hour at a constant current of 350 mA. For the detection of Brm, a polyclonal anti-Brm antibody (1:500) was used [88]. The appropriate secondary antibody (GE Healthcare, UK) was used at a dilution of 1:2000. GAPDH antibody (Genetex Inc., Irvine, CA, USA) was used as the loading control. Western blots were developed with an ECL Western blot detection kit (GE Healthcare, UK).

## SUPPLEMENTARY TABLE


