# Supplementary material for: Induction of functional Brm protein from Brm knockout mice
Source: Oncoscience. 2015 Apr 18;2(4):349–61. doi: 10.18632/oncoscience.153 (PMC4468321; doi:10.18632/oncoscience.153)
Supplement: Supplementary file 1 [file oncoscience-02-0349-s001.pdf]

Induction of functional Brm protein from Brm knockout mice

Supplementary Material

Supplemental table 1: qPCR primers

Gene	Forward primer (5')	Reverse primer (3')
Brm	5'-GCAGGAACGAGAGTACAGACTT-3'	5'-CGGTTGCTTTGGTGCGTAAA-3'
Gapdh	5'-CACTCTTCCACCTTCGATGC-3'	5'-GGGTGGTCCAGGGTTTCTTA-3'
DDX58	5'-AATATTCTGATTGCCACCTCAGTT-3'	5'-CACCAGCATTACTAGTCAGAAGGA-3'
P8	5'-ATAGCCTGGCCCATTCCTAC-3'	5'-CCTCGCTTCTTCCTCTCTGA-3'
LGAL3	5'-CATACTGGGAGAGGCTTCTGG	5'-CCCACTGGCCTCTGTAGAAGAT-3'
CEACAM-1	5'-CCCAGAATGACACAGGATTC-3'	5'-CCACCACAGGTAGGTTGTGT-3'
BST2	5'-AAGAAAGTGGAGGAGCTTGAGG-3'	5'-CGCTCAGAACTGATGAGATCAA-3'
